# Evaluation of renal cold ischemia–reperfusion injury with intravoxel incoherent motion diffusion-weighted imaging and blood oxygenation level-dependent MRI in a rat model

**DOI:** 10.3389/fphys.2023.1159741

**Published:** 2023-05-22

**Authors:** Yan Ren, Lihua Chen, Yizhong Yuan, Jipan Xu, Fangjie Xia, Jinxia Zhu, Wen Shen

**Affiliations:** ^1^ Department of Radiology, Tianjin First Central Hospital, Tianjin Institute of Imaging Medicine, Tianjin, China; ^2^ Department of Radiology, Tianjin Medical University General Hospital, Tianjin, China; ^3^ Department of Radiology, Tianjin Institute of Imaging Medicine, Tianjin Medical University First Central Hospital, Tianjin, China; ^4^ MR Collaborations, Siemens Healthcare Ltd., Beijing, China

**Keywords:** cold ischemia, reperfusion injury, IVIM, BOLD, kidney

## Abstract

**Purpose:** Cold ischemia-reperfusion injury (CIRI) is one of the most serious complications following renal transplantation. The current study investigated the feasibility of Intravoxel Incoherent Motion (IVIM) imaging and blood oxygenation level-dependent (BOLD) in the evaluation of different degrees of renal cold ischemia-reperfusion injury in a rat model.

**Methods:** Seventy five rats were randomly divided into three groups (N = 25 for each group): T0: sham-operated group, T2/T4: CIRI groups with different cold ischemia hours (2, 4 h, respectively). The rat model of CIRI group was established by left kidney cold ischemia with right nephrectomy. All the rats received a baseline MRI before the surgery. Five rats in each group were randomly selected to undergo an MRI examination at 1 h, day 1, day 2 and day 5 after CIRI. The IVIM and BOLD parameters were studied in the renal cortex (CO), the outer stripe of the outer medulla (OSOM), and the inner stripe of the outer medulla (ISOM) followed by histological analysis to examine Paller scores, peritubular capillary (PTC) density, apoptosis rate and biochemical indicators to obtain the contents of serum creatinine (Scr), blood urea nitrogen (BUN), superoxide dismutase (SOD) and malondialdehyde (MDA).

**Results:** The D, D*, PF and T2* values in the CIRI groups were lower than those in the sham-operated group at all timepoints (all *p* < 0.05). The prolonged cold ischemia times resulted in gradually lower D, D*, PF and T2* values (all *p* < 0.05). The D and T2* values of cortex and OSOM in Group T0 and T2 returned to the baseline level (all *p* > 0.05) except Group T4. The D* and PF values of cortex, OSOM and ISOM in Group T2 and T4 still remained below the normal levels (all *p* < 0.05) except Group T0. D, D*, PF and T2* values were strongly correlated with histopathological (Paller scores, PTC density and apoptosis rate) and the biochemistry indicators (SOD and MDA) (|r|>0.6, *p* < 0.001). D*, PF and T2* values were moderately to poorly correlated with some biochemistry indicators (Scr and BUN) (|r|<0.5, *p* < 0.05).

**Conclusion:** IVIM and BOLD can serve as noninvasive radiologic markers for monitoring different degrees of renal impairment and recovery after renal CIRI.

## Introduction

Renal cold ischemia-reperfusion injury (CIRI) is one of the most serious complications following renal transplant (Dugbartey et al., 2021). CIRI has a higher incidence in patients, potentially increasing the risk of acute kidney injury (AKI), chronic kidney disease and transplant failure. The life quality of patients are seriously influenced and the rejection risk of transplanted kidney is conspicuously increased (Hart et al., 2017). The complicated pathological process was initiated which gives rise to decreased blood flow, hypoxia and cellular damage (Zhang W. et al., 2018). However, CIRI can be reversible at early stage, thus it is essential to monitor the progression for improving outcomes (Francis and Baynosa, 2017).

The puncture biopsy is considered as the gold standard to assess kidney injury. Nevertheless, biopsy is invasive and prone to sampling errors (Aaltonen et al., 2020). The serum creatinine (Scr), blood urea nitrogen (BUN) and glomerular filtration rate (GFR) are regularly used to evaluate kidney function. However, these indices are affected by multiple factors and can not be applied to evaluate the dysfunction for individual kidney (Khawaja et al., 2019). For the above reasons, the biopsy and biochemistry indicators were not suitable as routine approaches for dynamic detection of renal CIRI.

Previous several studies, whether contrast-enhanced computed tomography (CT) (Braunagel et al., 2016) or ultrasound (US) (Ryan et al., 2020), are confined to the resolution of images, character of operator dependent and iodine-based contrast media. Multiparametric MRI is a current useful technique for evaluating the tissue microenvironment alternations including structure and function information. To date, most prior studies focused on exploring the mechanisms associated with MRI methods in warm ischemia reperfusion injury (Ko et al., 2017; Zhang et al., 2021). In clinical practice, while, cold ischemia duration commonly lasts longer than warm ischemia (Hart et al., 2017). It is therefore necessary to concentrate on renal cold ischemia reperfusion injury, which is quite significant for preclinical assessment in order to improve the success of surgery and reduce complications.

Multiparametric MRI offers the potential to quantitatively and noninvasively detect renal tissues of ischemia and hypoxia impairment post CIRI. Intravoxel Incoherent Motion diffusion-weighted imaging (IVIM) employs a bi-exponential model to estimate both water molecule diffusion and microcirculation simultaneously, providing measures of the true water molecule diffusion coefficient (D), the perfusion-related diffusion coefficient (D*), and the perfusion fraction (PF). (Le Bihan D et al., 1986). Blood oxygenation level-dependent (BOLD) utilized deoxyhemoglobin to evaluate the blood oxygenation level in tissue by magnetic susceptibility alternations and the provided R2* measurement was sensitive to blood oxygenation, blood flow, and blood volume (Zhao et al., 2021). Although BOLD are currently used to assess renal function in various kidney diseases (Pedersen et al., 2010; Hu et al., 2014), evaluation of renal CIRI has been limited. A previous study (Rheinheimer et al., 2012) has characterized the IVIM MRI measurements on the transplanted kidney of different cold preservation time, however, histopathological and immunohistochemistry results have not obtained. Our present study expanded the scope work on renal CIRI in a dynamic and time-dependent way to explore the micropathologic progression by MRI.

Consequently, the purpose of our study was to determine IVIM and BOLD measurements changes of renal CIRI in a rat model and to compare the quantitative measurements with renal histology and biochemical indicators.

## Material and methods

### Experimental design and animal models

**FIGURE 1:**
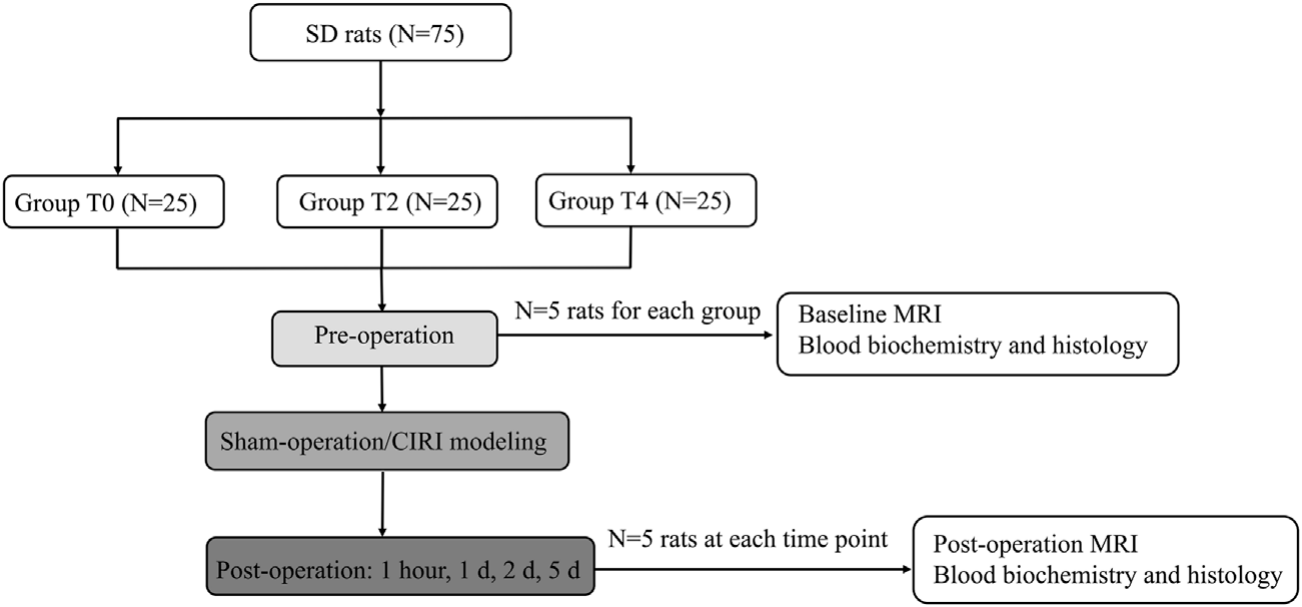
Flowchart of the experimental procedure.

This study was approved by Ethics Committee of Nankai University. All researchers strictly adhered to the animal ethical guidelines in performing experiments. The 75 healthy male Sprague Dawley (SD) rats weighing between 200 g to 250 g at 8–10 weeks of age were purchased from the National Institutes for Food and Drug Control. The rats were maintained in a room at a temperature of 24°C–26°C and a humidity of 50%–60%. The rats were housed under conventional conditions with a 12-h light/dark cycle and had free access to standard feed and drinking water. All the rats were randomly divided into three groups (N = 25 for each group), T0: sham-operated group, T2: 2-h cold ischemia group, T4: 4-h cold ischemia group. All the rats received a baseline MRI before the surgery. Five rats in each group were randomly selected to undergo an MRI examination at 1 h, day 1, day 2, day 5 after CIRI (Figure 1).

Cold ischemia reperfusion injury was induced by the following steps (Wei et al., 2019). The rats were anesthetized using 2% isoflurane with a flow rate of 0.5 L/min during operation. First, the abdominal cavity was opened through a middle abdominal incision to expose the left kidney and aorta. The exposed intestines were covered with saline-soaked gauze. The left adrenal vein and gonad vein were dissociated and ligated. The left renal pedicle, the left renal artery and vein, the upper and lower end of abdominal aorta were exposed and dissociated. Then, the non-traumatic microvascular clamps were used in the above locations to induce ischemia. A small slit was cut on the left renal vein. The Ringer’s solution at 0°C–4°C was injected using a 24G needle until the solution from the renal venous slit and the left kidney color became pale. The left renal artery and left renal pedicle were pinched and the clamps of the abdominal aorta were removed to restore the blood flow. The ice saline gauze was placed on the dorsal side of the kidney. The ice debris was placed evenly around the kidney and replenished at any time to maintain low temperature during the cold ischemia period for 2 and 4 h, respectively. Then the clamps were removed, and the left kidney blood flow was restored. Finally the right kidney was removed and the abdomen cavity was closed. The sham-operated group (Group T0) only performed laparotomy and resection of right kidney.

### MRI protocol

MRI scans were performed on a 3T MRI system (MAGNETOM Prisma; Siemens Healthcare, Erlangen, Germany) with an 8-channel experimental animal coil (Shanghai Chenguang Medical Technology Co., Ltd., Shanghai, China). The rats were anesthetized using 5% isoflurane with a flow rate of 1 L/min during scanning. In addition, we used usual air and the oxygen fraction was approximately 21%.

A coronal T2-weighted turbo spin-echo (TSE) sequence was performed for morphological evaluation of the kidneys using a repetition time/echo time (TR/TE) of 4,120/100 ms, a field of view (FOV) of 100 × 75 mm^2^, a slice thickness of 1.5 mm, a matrix of 192 × 192, a reconstructed voxel size of 0.3 × 0.3 × 1.5 mm^3^ and an acquisition time of 4 min and 13 s.

IVIM MRI was acquired in the coronal plane using a integrated shimming (iShim) multi b-value single-shot diffusion-weighted echo planar imaging sequence with free breathing. The parameters were as follows: a TR/TE of 2,300/74 ms, a FOV of 140 × 114 mm^2^, a slice thickness of 3.0 mm, a matrix of 120 × 98 and a reconstructed voxel size of 0.6 × 0.6 × 3.0 mm^3^. Ten b-values (0, 10, 20, 30, 50, 100, 200, 300, 500 and 800 s/mm^2^) were obtained in three diffusion gradient directions. The acquisition time of IVIM DWI was 7 min and 9 s.

BOLD MRI was performed in an coronal T2* map using a multiple gradient echo sequence. The parameters were as follows: TR of 2500 ms, 6 TEs of 3.22, 5.83, 8.42, 11.01, 13.63, 16.22 ms, FOV of 85 × 62 mm^2^, a slice thickness of 3.0 mm, a matrix of 160 × 160 and a reconstructed voxel size of 0.5 × 0.5 × 3.0 mm^3^. The acquisition time of BOLD MRI was 3 min and 57 s.

### Image analysis

**FIGURE 2:**
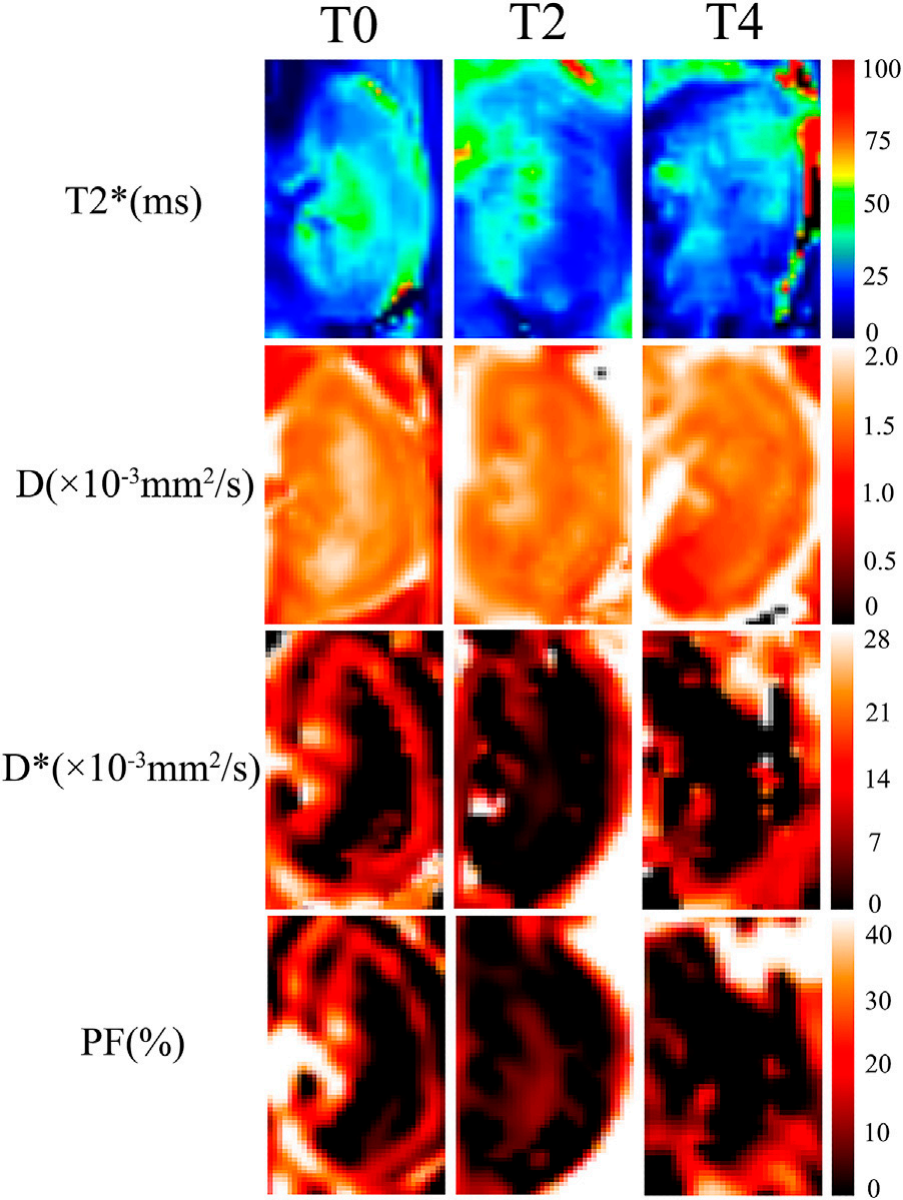
Representative images of IVIM and BOLD parametric maps in Group T0, T2 and T4 on day 1. The D, D*, PF and T2* maps showed lower values at 1 h than those on day 5. As cold ischemia time prolonged, the parametric maps showed gradually decreased signals from Group T0 to T4 (the length ratio reference, 1:1).

The IVIM-derived parameters, pure molecular diffusion (D), pseudo-diffusion (D*), and the perfusion fraction (PF), were calculated by using a prototype software (MR Body Diffusion Toolbox, Siemens Healthcare, Erlangen, Germany) based on the bi-exponential model: S(b)/S (0)=(1- f) × exp (- b ×D) + f× exp (- b × D^*^). S(b) is the signal intensity of the tissue within the diffusion gradient field and S (0) is the signal intensity of the tissue without the diffusion gradient field. BOLD-derived parameters,T2* values were acquired from multi-echo gradient echo images (Figure 2).

**FIGURE 3:**
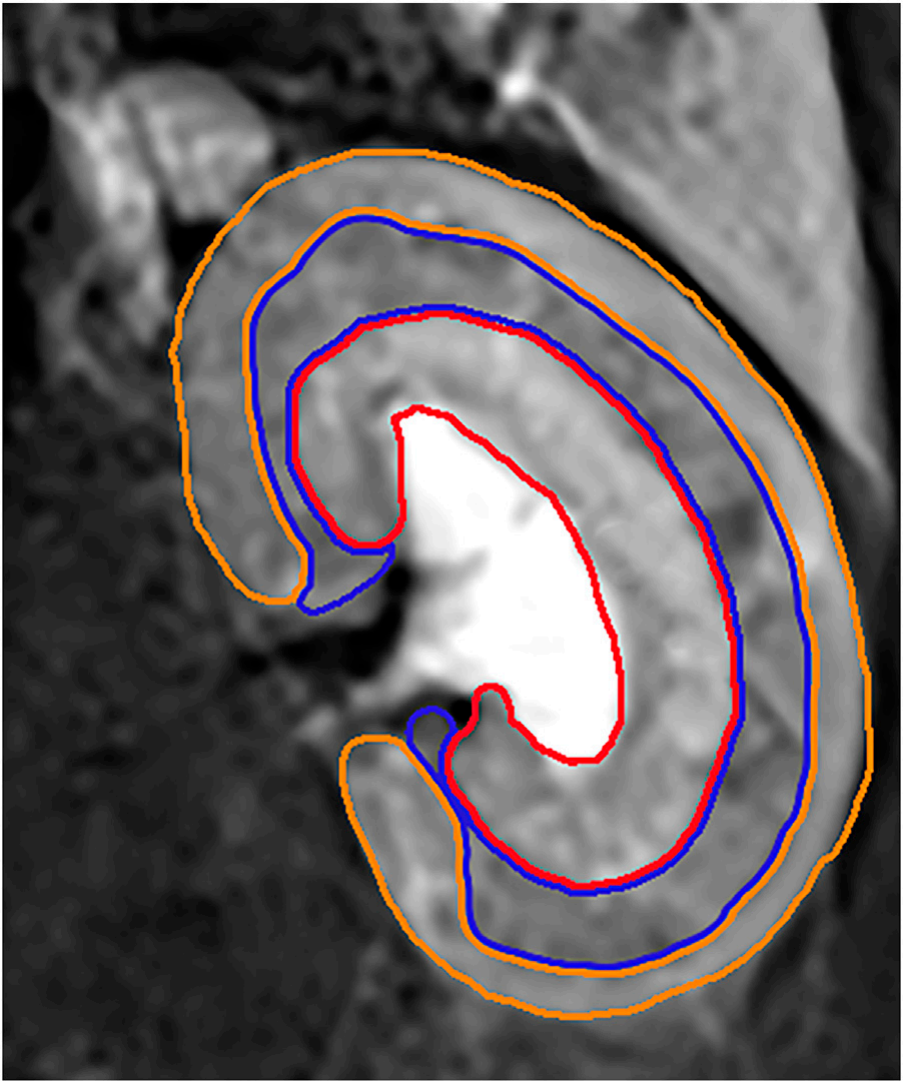
The orange ROI represented the cortex region. The blue ROI represented the OSOM region. The red ROI represented the ISOM region.

IVIM and BOLD MRI data were assessed blindly by two specialist radiologists (L.C. and Y.R. with 15 and 6 years of experience in abdominal MRI, respectively). The regions of interest (ROI) were positioned in the cortex (CO), the outer stripe of the outer medulla (OSOM), and the inner stripe of the outer medulla (ISOM). The cortex includes glomerulus, the proximal and distal convoluted tubule segments. The OSOM consists of the S3 segment of the proximal tubule containing much endoplasmic reticulum and the highest brush border of all kidney cells. The ISOM contains the distal straight tubules, some collecting ducts, and loops of Henle. The largest section of kidney was selected and the renal cortex, OSOM, and ISOM region were outlined manually with T2-weighted images as a reference. The voxels in the ROIs were averaged for evaluation based on the non-linear fitting method. (Figure 3).

### Blood biochemical index measurement

After the acquisition of the MRI scanning, 4 mL blood samples were extracted through the abdominal aorta and centrifuged at 3,000 r/min. The following samples were analyzed: serum creatinine (Scr), blood urea nitrogen (BUN), superoxide dismutase (SOD) and malondialdehyde (MDA).

### Histopathological and immunohistochemistry analyses

The left kidneys were removed from renal hilum and stored in 4% paraformaldehyde solution. The renal tissues were sectioned for hematoxylin and eosin staining to analyze the pathomorphological characteristics of the kidneys. The renal tubule injury score was evaluated by the Paller’s standard (PALLER et al., 1984). Peritubular capillary (PTC) density was stained with CD34 (endothelial cell-specific marker) expression and were counted in ten randomly chosen microscopic fields on each section in kidney interstitium and capillary density was presented as the average number of capillaries/mm^2^ (Ma et al., 2010). Apoptotic cells marking were performed with TUNEL (TdT-mediated biotin-16-dUTP nick-end labeling) staining. Kidney sections were analyzed at a magnification of ×200 in light microscope. The percentage of apoptotic cells (number of apoptotic cells/total number of cells * 100) was calculated as the apoptosis rate (%).

### Statistical analysis

Statistical analysis was performed using the statistical package for social science (SPSS) software version 26.0 (SPSS Inc., Chicago, IL, United States), and all measured parameters were presented as mean ± standard deviation (SD). The Shapiro–Wilk test was applied to assess normally distributed data. The intraclass correlation coefficient (ICC) was used to determine the interobserver agreement of IVIM parameters, with ICC <0.50, 0.50≤ ICC <0.75, 0.75≤ ICC <0.90, ICC ≥0.90 indicating poor, moderate, good and excellent agreement, respectively (de Vet et al., 2006). Repeated-measures ANOVA was used to compare IVIM-derived parameters and T2* values in each group at different time points. The multivariate analysis of variance was used to compare MRI parameters between different groups at each time point. Spearman correlation coefficients were calculated to determine the correlation between MRI parameters and histologic and blood biochemical indices. The degree of correlation was defined as poor or no correlation (0 ≤ r < 0.25), fair correlation (0.25 ≤ r < 0.5), moderate to good correlation (0.5 ≤ r < 0.75) and very good to excellent correlation (r ≥ 0.75) (S et al., 2014). *P* < 0.05 was considered statistically significant. The GraphPad Prism Inc. software version 8.0 was used to plot the results.

## Results

### ICC analyses

The D, D*, PF and T2* values in the CO showed excellent interobserver agreement with ICCs of 0.953 (0.887–0.980), 0.841 (0.505–0.940), 0.958 (0.796–0.986), and 0.920 (0.826–0.964) respectively. The D, D*, PF and T2* values in the OSOM showed great interobserver agreement with ICCs of 0.881 (0.750–0.946), 0.920 (0.828–0.964), 0.880 (0.748–0.945), and 0.928 (0.845–0.968) respectively. The D, D*, PF and T2* values in the ISOM showed excellent interobserver agreement with ICCs of 0.887 (0.761–0.949), 0.868 (0.724–0.940), 0.903 (0.792–0.956), and 0.916 (0.820–0.962) respectively. (Supplementary Material Table S1).

### IVIM parameters analyses

**FIGURE 4:**
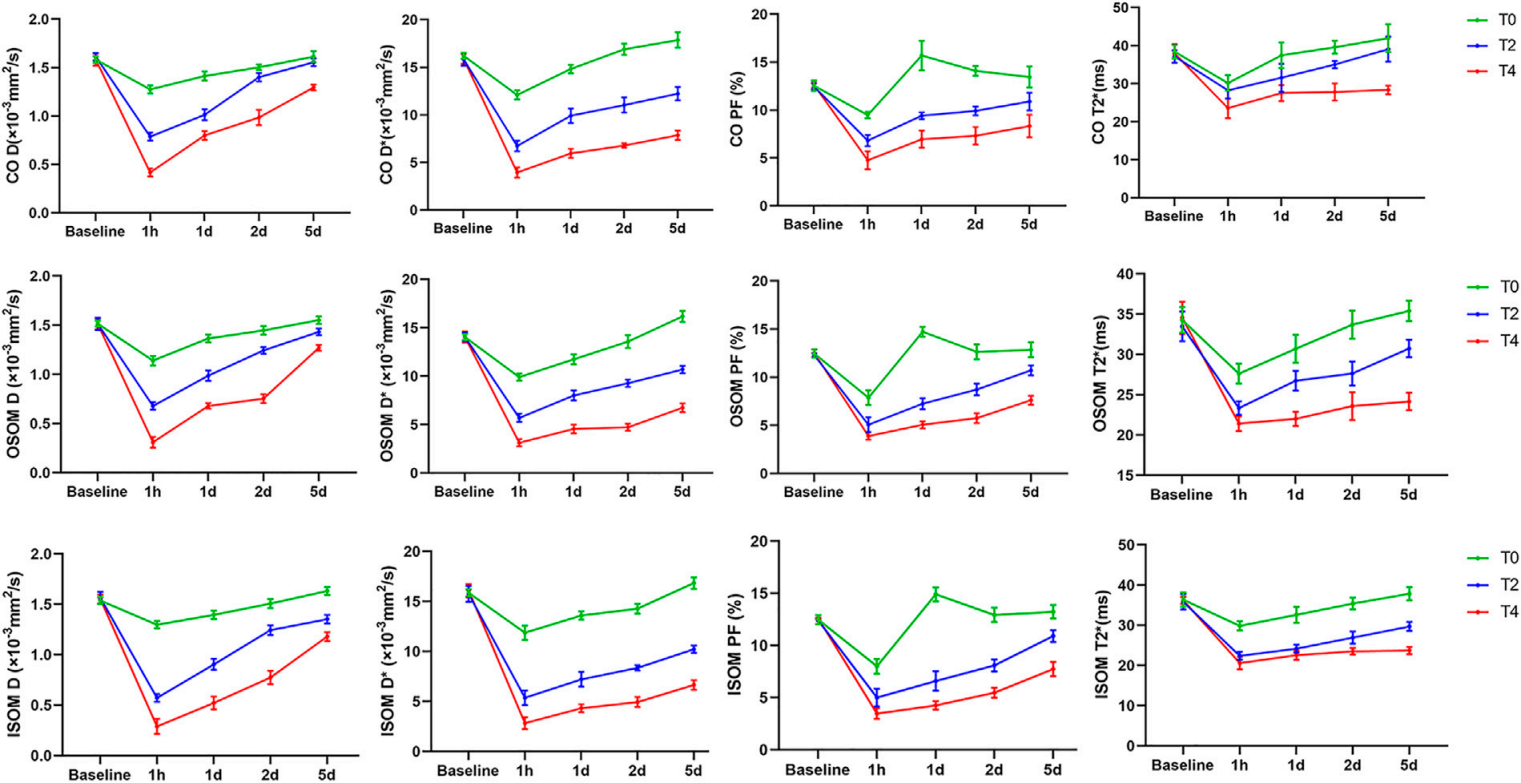
The temporal changes of IVIM-derived D, D*, PF and BOLD-derived T2* values before and after operation. The values of all groups in the different regions exerted a sharp decrease followed by a gradual increase. CO, cortex; OSOM, the outer stripe of the outer medulla; ISOM, the inner stripe of the outer medulla 1h, 1 hour; 1d, day 1; 2d, day 2; 5d, day 5; D, pure molecular diffusion; D*, pseudo-diffusion; PF, perfusion fraction.

The IVIM parameters on the kidney over time in the Group T0,T2, T4 are summarized in Table 1. At 1 h after CIRI, the values of all parameters decreased to the lowest level (*p* < 0.05) and then gradually increased. Prolonged cold ischemia times resulted in gradually lower D, D* and PF values at all the operative time points. D values dropped dramatically at 1 h post-operation in all groups (*p* < 0.05). In the Group T0, D values returned to the baseline levels on day 2. In the Group T2, D values gradually increased to the baseline levels on day 2 in the cortex and day 5 in the OSOM and ISOM, respectively (*p* < 0.05). However, D values of all regions in the Group T4 remained decreased than Group T0 and T2 (*p* < 0.001) and did not return to the baseline on day 5 (Figure 4; Table 1).

**Table 1:** Comparison of MRI parameters from sham-operated and CIRI groups of CO,OSOM and ISOM at different timepoints.

CO Time	D(×10^-3^mm^2^/s)			*Pvalue*	D*(×10^-3^mm^2^/s)			*Pvalue*	PF(%)			*P value*	T2*(ms)			*Pvalue*
	T0	T2	T4		T0	T2	T4		T0	T2	T4		T0	T2	T4	
Baseline	1.58 ±0.03	1.61 ±0.04	1.58 ±0.06	0.514	16.32±0.32	15.87 ±0.68	15.90 ±0.52	0.498	12.55±0.53	12.47 ±0.31	12.65 ±0.36	0.784	38.42±1.77	37.19 ±1.63	37.96 ±2.43	0.620
1h	1.28±0.04*	0.79±0.04**	0.42±0.04**	<0.001	12.11±0.47*	6.74±0.55**	3.95±0.52**	<0.001	9.48±0.32*	6.82±0.58*	4.77±0.94*	<0.001	30.20±2.07*	28.26±2.12*	23.60±2.61*	0.002
1d	1.41±0.05*	1.01±0.06**	0.80±0.04**	<0.001	14.84±0.44	9.92±0.76*	5.96±0.47**	<0.001	15.70±1.54*	9.42±0.36*	6.96±0.47*	<0.001	37.48±3.31	31.60±3.64	27.55±2.10*	0.001
2d	1.50±0.03	1.40±0.04	0.99±0.08*	<0.001	16.91±0.60	11.04±0.79*	6.80±0.22**	<0.001	14.10±0.51*	9.92±0.46*	7.33±0.90*	<0.001	39.63±1.68	35.06±0.93	27.84±2.25*	<0.001
5d	1.61±0.06	1.56±0.04	1.30±0.03*	<0.001	17.88±0.81	12.24±0.71*	7.88±0.48**	<0.001	13.45±1.10	10.90±0.91*	8.34±1.17*	<0.001	41.97±3.63	39.11±3.29	28.38±1.11*	<0.001
*Fvalue*	47.747	276.33	312.877		75.43	140.089	563.325		30.468	58.648	44.891		13.008	15.655	33.971	
*P value*	<0.001	<0.001	<0.001		<0.001	<0.001	<0.001		<0.001	<0.001	<0.001		<0.001	<0.001	<0.001	
**OSOM**	**D(×10** ^**-3**^ **mm** ^**2**^ **/s)**			***P value***	D*(×10 ^**-3**^ **mm** ^**2**^ **/s)**			***P value***	**PF(%)**			***P value***	T2*(ms)			***P value***
**Time**	**T0**	**T2**	**T4**		**T0**	**T2**	**T4**		**T0**	**T2**	**T4**		**T0**	**T2**	**T4**	
Baseline	1.52 ±0.03	1.51±0.06	1.51±0.05	0.992	14.04±0.30	14.07±0.46	14.06 ±0.58	0.997	12.46±0.44	12.27±0.22	12.51±0.38	0.552	34.28±1.58	33.47±1.84	34.54±1.99	0.634
1h	1.14±0.05*	0.68±0.04*	0.31±0.05**	<0.001	9.90±0.36**	5.70±0.43**	3.13±0.36**	<0.001	7.88±0.76*	5.08±0.76*	3.88±0.36**	<0.001	27.62±1.24*	23.35±0.84*	21.48±0.93*	<0.001
1d	1.37±0.04*	0.99±0.05*	0.68±0.03**	<0.001	11.73±0.52**	8.01±0.52**	4.55±0.44**	<0.001	14.73±0.51*	7.25±0.56*	5.07±0.36**	<0.001	30.70±1.72*	26.74±1.23*	22.01±0.87*	<0.001
2d	1.45±0.04	1.24±0.03*	0.75±0.05*	<0.001	13.57±0.67	9.27±0.38**	4.73±0.37**	<0.001	12.64±0.77	8.73±0.60*	5.76±0.52**	<0.001	33.70±1.76	27.64±1.49*	23.60±1.72*	<0.001
5d	1.55±0.03	1.43±0.03	1.27±0.03*	<0.001	16.18±0.57	10.7±0.37*	6.75±0.45**	<0.001	12.85±0.76	10.73±0.52*	7.62±0.46**	<0.001	35.40±1.28	30.74±1.08	24.15±1.08*	<0.001
*F value*	82.381	235.043	590.746		129.135	333.855	545.242		71.065	127.953	280.361		20.352	43.045	74.863	
*P value*	<0.001	<0.001	<0.001		<0.001	<0.001	<0.001		<0.001	<0.001	<0.001		<0.001	<0.001	<0.001	
**ISOM**	**D(×10** ^**-3**^ **mm** ^**2**^ **/s)**			***P value***	D*(×10 ^**-3**^ **mm** ^**2**^ **/s)**			***P value***	**PF(%)**			***P value***	T2*(ms)			***P value***
**Time**	**T0**	**T2**	**T4**		**T0**	**T2**	**T4**		**T0**	**T2**	**T4**		**T0**	**T2**	**T4**	
Baseline	1.54 ±0.03	1.56±0.06	1.55±0.04	0.691	15.85±0.34	15.75±0.71	16.10 ±0.66	0.637	12.47±0.44	12.39±0.13	12.59±0.05	0.515	36.37±1.76	35.83±1.93	36.18±0.88	0.863
1h	1.30±0.04*	0.57±0.04**	0.29±0.07**	<0.001	11.87±0.71*	5.38±0.72**	2.86±0.58**	<0.001	8.00±0.70*	5.01±0.84*	3.40±0.49**	<0.001	29.87±1.14*	22.41±0.97*	20.60±1.57**	<0.001
1d	1.40±0.04*	0.90±0.06*	0.52±0.06**	<0.001	13.59±0.52*	7.21±0.74**	4.32±0.39**	<0.001	14.91±0.66*	6.60±0.92*	4.25±0.40**	<0.001	32.62±1.97*	24.17±0.99*	22.55±1.15*	<0.001
2d	1.51±0.04	1.24±0.05*	0.77±0.07*	<0.001	14.28±1.41	8.36±0.24**	4.95±0.49**	<0.001	12.92±0.68	8.08±0.58*	5.46±0.49**	<0.001	35.40±1.47	26.92±1.51*	23.51±0.79**	<0.001
5d	1.63±0.03	1.35±0.04	1.19±0.06*	<0.001	16.83±0.58	10.22±0.38*	6.65±0.50**	<0.001	13.22±0.64	10.90±0.57*	7.74±0.69*	<0.001	37.86±1.67	29.71±1.09*	23.52±1.03*	<0.001
*F value*	45.79	262.790	278.806		62.046	243.452	622.137		93.499	89.421	270.632		19.777	68.409	147.251	
*P value*	<0.001	<0.001	<0.001		<0.001	<0.001	<0.001		<0.001	<0.001	<0.001		<0.001	<0.001	<0.001	

*P < 0.05 versus baseline.

**P < 0.001 versus baseline.

CO, cortex; OSOM, the outer stripe of the outer medulla; ISOM, the inner stripe of the outer medulla; 1h, 1 hour; 1d, day 1; 2d, day 2; 5d, day 5.

D* values decreased significantly at 1 h then significantly increased from day 1 to day 5 in all groups (*p* < 0.05). In the Group T0, the D* values of cortex, OSOM and ISOM returned to the baseline levels on day 1 and day 2, respectively. In the Group T2 and T4, the D* values were significantly lower compared with the Group T0 at all postoperative time points (*p* < 0.001) and still below the baseline on day 5. (Figure 4; Table 1).

PF values declined significantly at 1 h followed by sharply increase to exceed baseline level on day 1 and day 2 in Group T0 (*p* < 0.05). In the Group T2 and T4, the D* values were significantly lower compared with the Group T0 at all time points (*p* < 0.001) and still below the baseline on day 5 (Figure 4; Table 1).

### BOLD parameter analyses

T2* values of all groups decreased to the lowest level (*p* < 0.05) and then gradually increased. In the Group T0, T2* values returned to the baseline level on day 1 in cortex and day 2 in OSOM and ISOM. In the Group T2, T2* values of cortex and OSOM elevated to the baseline levels on day 1,5 respectively (*p* < 0.05), while T2* values of ISOM still below the normal level. In the Group T4, the T2* values were significantly lower than those of the Group T0 at all postoperative time points (*p* < 0.05) and did not return to the normal level on day 5. (Figure 4; Table 1).

### Blood biochemical analyses

#### Serum creatinine

Scr in the Group T0 showed no obvious changes after operation. In the Group T2 and T4, Scr increased to the top levels on day 2 but remained elevated above the baseline (*p* < 0.05) (Table 2).

**Table 2:** Changes in blood biochemical findings from sham-operated and CIRI groups at different timepoints.

**Time**	**Scr(μmol/L)**			BUN(mmol/L)			**SOD (U/ml)**			**MDA (nmol/ml)**		
	**T0**	**T2**	**T4**	**T0**	**T2**	**T4**	**T0**	**T2**	**T4**	**T0**	**T2**	**T4**
Baseline	56.74±10.78	56.04±10.55	53.94±9.08	8.45±1.64	8.99±1.60	9.24±1.32	249.43±24.75	239.32±9.51	254.07±12.46	3.04±0.51	3.38±0.62	3.44±0.58
1h	47.25±7.25	59.62±17.29	59.34±6.37	9.90±0.76	8.45±1.44	8.16±0.62	138.62±8.88*	134.39±6.03*	120.73±3.54**	4.01±0.92	14.05±1.15*	14.81±0.90**
1d	52.40±6.79	86.98±9.61	98.96±7.34*	9.22±1.21	11.46±0.90	15.86±1.17*	140.75±8.27*	124.94±6.52**	110.78±7.51**	3.80±0.46	14.38±2.76*	18.71±2.30*
2d	47.74±10.78	100.20±7.50*	109.24±3.29*	9.69±1.51	12.03±0.71*	17.88±1.45*	145.24±7.35*	125.89±2.80**	114.20±4.62**	4.84±0.60	16.99±4.24*	18.95±2.08*
5d	58.17±10.01	97.64±7.89*	106.56±12.58*	10.45±0.78	12.77±1.20	20.95±1.79*	155.05±3.35*	147.64±5.15*	122.35±7.02**	3.01±0.40	15.51±3.94*	14.14±2.76*

*P < 0.05 versus baseline.

**P < 0.001 versus baseline.

1h, 1 hour; 1d, day 1; 2d, day 2; 5d, day 5; Scr, serum creatinine; BUN, blood urea nitrogen;SOD,superoxide dismutase;MDA, malondialdehyde.

#### Blood urea nitrogen

In the Group T0, BUN showed no obvious changes after operation. In the Group T2 and T4, BUN were not significantly different from the baseline at 1 h but progressively increased from day 1–5. The BUN in the Group T4 were greater than those in the Group T0 on day 1,2 and 5 (*p* < 0.05) (Table 2).

#### Superoxide dismutase

In the Group T0 and T2, the SOD values dramatically dropped at 1 h (*p* < 0.05) then gradually increased while the Group T4 continued to decrease on day 1. The SOD values of all groups remained below the normal level until day 5. At all timepoints, the SOD values of Group T4 displayed significantly different compared with Group T0 and T2 (all *p* < 0.05). The SOD values of Group T2 were significantly different from the Group T0 on day 1 and day 2 (all *p* < 0.05) (Table 2).

#### Malondialdehyde

In the Group T0, the MDA values were not significantly different after operation (*p* > 0.05). In the Group T2 and T4, the MDA values obviously elevated at 1 h (*p* < 0.05) and progressively increased to the top on day 2 (all *p* < 0.001). At all post-operation timepoints, the MDA values of Group T0 demonstrated significantly different compared with the Group T2 and T4 (all *p* < 0.001) (Table 2).

### Histologic analyses

#### Paller score

**FIGURE 5:**
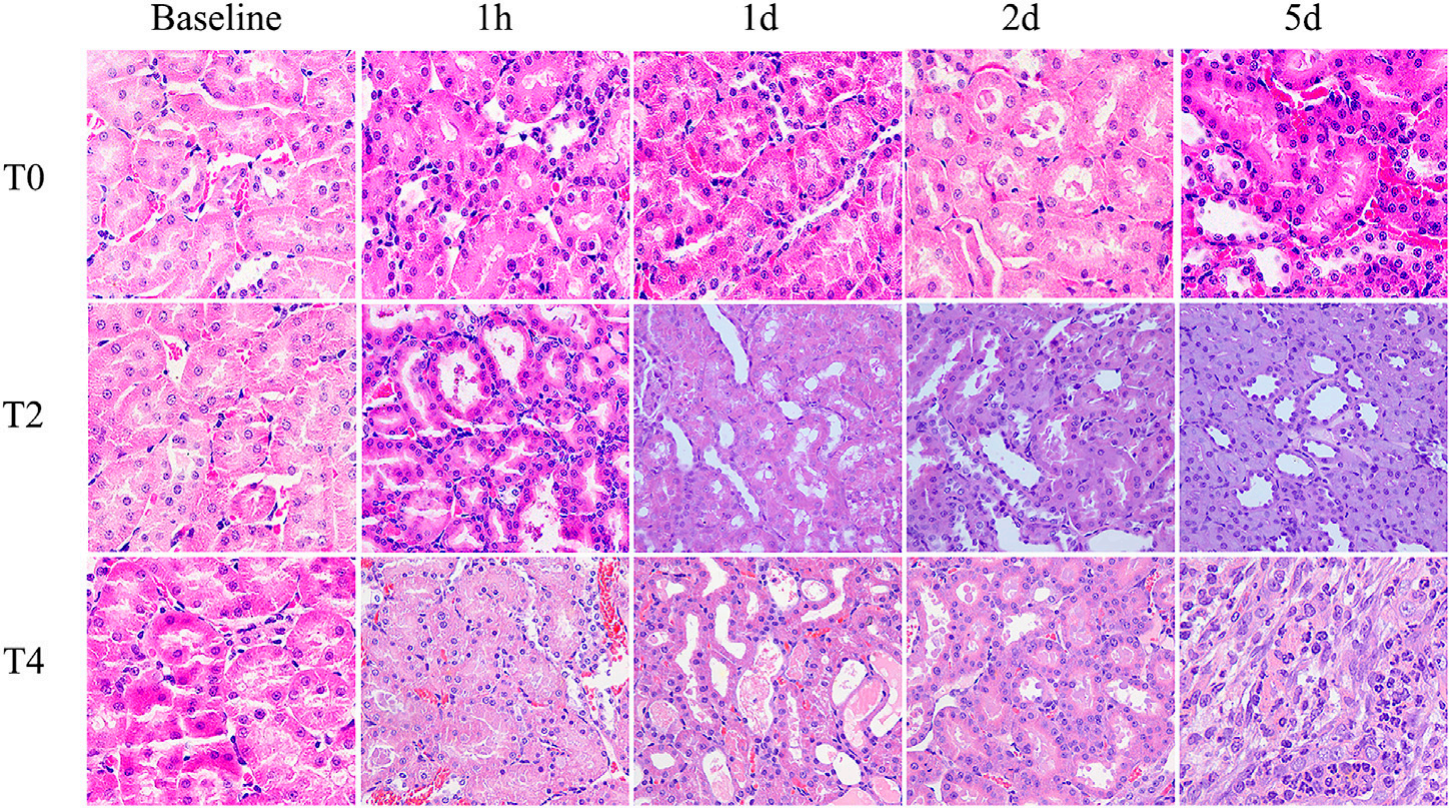
Histological pathology of kidney with HE staining (×400) from the Group T0, T2 and T4. For each timepoint after CIRI, the damage degree of renal tubular epithelial cells in the Group T2 and T4 were more severe than those observed in Group T0: renal tubules dilation, exfoliated cells, brush border desquamation as well as interstitial tissue edema. Tubular necrosis and cellular detachment with tubular cell casts were evident in T2 and T4 groups at day 1 and day 2.

Paller score rose significantly at 1 h and gradually declined from day 1–5. In the Group T0, the score decreased back to the baseline by day 1. In the Group T2 and T4, Paller scores were significantly larger than the baseline and those of the Group T0 at all timepoints after CIRI (all *p* < 0.05) (Figure 5; Table 3).

**Table 3:** Changes in pathological and immunohistochemical findings from sham-operated and CIRI groups at different timepoints.

**Time**	**Paller Score**			**PTC Density(/mm** ^**2**^ **)**			**Apoptosis Rate(%)**		
	**T0**	**T2**	**T4**	**T0**	**T2**	**T4**	**T0**	**T2**	**T4**
Baseline	15.20±1.10	16.00±2.65	18.00 ±2.92	476.40±4.83	481.60±12.03	480.40±7.50	0.59±0.09	0.51±0.10	0.57±0.07
1h	24.8±3.96*	46.20±2.49**	56.8±7.66*	417.00±12.51*	375.00±5.74*	319.80±18.24*	0.97±0.18	2.74±0.34**	2.49±0.19**
1d	21.2±6.3	44.60±3.91*	68.2±10.43*	436.65±15.02*	402.31±8.73*	339.8±10.08**	1.42±0.30*	3.71±0.32**	4.60±0.46**
2d	20.2±7.05	42.00±7.97*	45.6±8.11*	445.20±14.01	410.80±10.13*	358.79±8.63**	1.26±0.26*	3.41±0.48*	4.39±0.38**
5d	16.8±4.49	40.20±3.03*	43.8±4.76*	469.20±21.17	431.60±5.46*	370.40±9.84**	0.77±0.31	1.54±0.32*	2.65±0.38*

*P < 0.05 versus baseline.

**P < 0.001 versus baseline.

1h, 1 hour; 1d, day 1; 2d, day 2; 5d, day 5; PTC, peritubular capillary.

### PTC density

**FIGURE 6:**
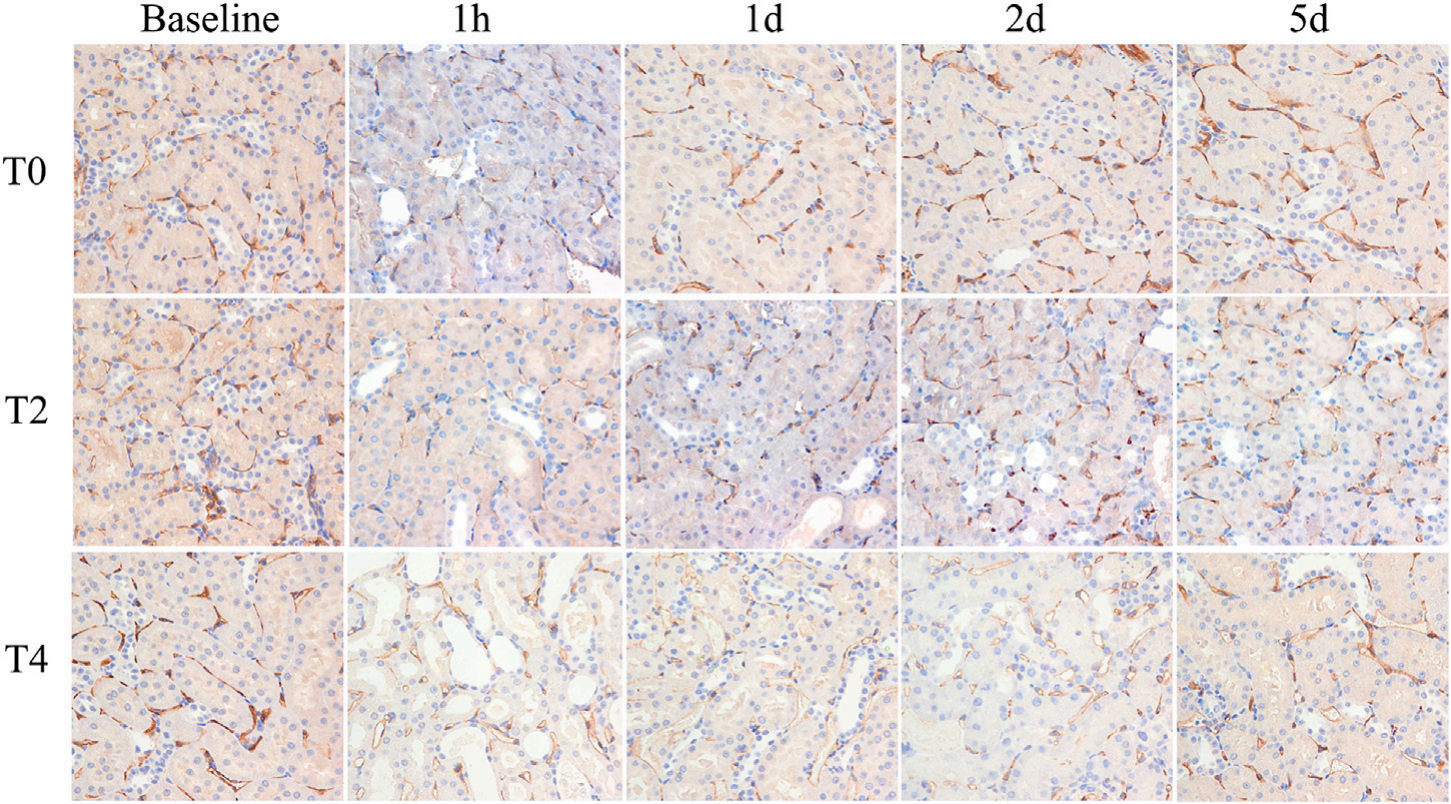
Histological pathology of kidney with CD34 staining (×400). Representative photomicrographs of CIRI groups at 1 h, 1 day, 2 days and 5 days. PTC was stained with CD34 expression and manifestated as brown color. PTC density decreased at 1 h then gradually increased on day 1. For each timepoint after CIRI, PTC density gradually decreased among T0, T2, and T4 groups as cold ischemia time prolonged.

PTC density reached the minimum at 1 h followed by increase on day 1 in all groups. In Group T0, the PTC density returned to the baseline by day 2. In Group T4, the PTC density were significantly lower than the baseline and those of the other groups at all postoperative time points. The PTC density in Group T0 were significantly higher than those of the Group T2 and T4 on day 1,2 and 5 (all *p* < 0.05) (Figure 6; Table 3).

### Apoptosis rate

**FIGURE 7:**
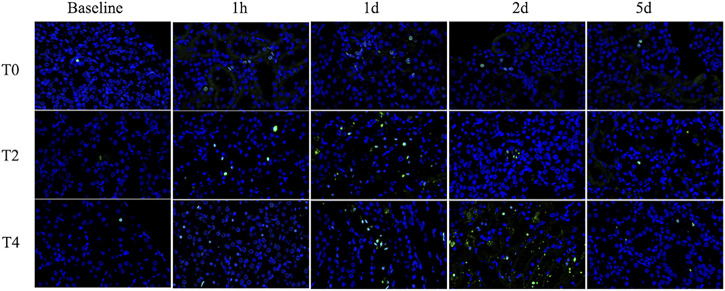
Immunohistochemistry pathology of kidney with TUNEL staining (×400). The cells labeled with green fluorescence were described as apoptotic cells and labeled with blue fluorescence were identified as the total cells. Representative photomicrographs showed that the apoptosis rate increased from 1 h to day 1 and then gradually decreased. For each timepoint after CIRI, the apoptosis rates of Group T2 and T4 were higher than those of Group T0.

Apoptosis rate increased significantly at 1 h and continuously peaked on day 1 followed by a mild decrease on day 2 in all groups. The apoptosis rate in the Group T0 returned to the baseline on day 5 while the Group T2 and T4 were still higher than the preoperation level (Figure 7; Table 3).

### Correlation between MRI parameters, histopathology, and biochemical indicators

In the kidney cortex, D, D*, PF and T2* were highly negatively correlated with Paller scores (r = −0.802, −0.877, −0.803, −0.754, respectively), positively correlated with PTC density (r = 0.901, 0.920, 0.847, 0.804, respectively), mildly negatively correlated with apoptosis rate (r = −0.759, −0.789, −0.665, −0.645, respectively).

In the kidney OSOM, D, D*, PF and T2* were highly negatively correlated with Paller scores (r = −0.833, −0.880, −0.836, −0.845, respectively), positively correlated with PTC density (r = 0.914, 0.939, 0.856, 0.911, respectively), mildly negatively correlated with apoptosis rate (r = −0.777, −0.826, −0.698, −0.819, respectively).

In the kidney ISOM, D, D*, PF and T2* were highly negatively correlated with Paller scores (r = −0.795, −0.900, −0.848, −0.875, respectively), positively correlated with PTC density (r = 0.886, 0.938, 0.845, 0.902, respectively), mildly negatively correlated with apoptosis rate (r = −0.788, −0.848, −0.717, −0.809, respectively).

**FIGURE 8:**
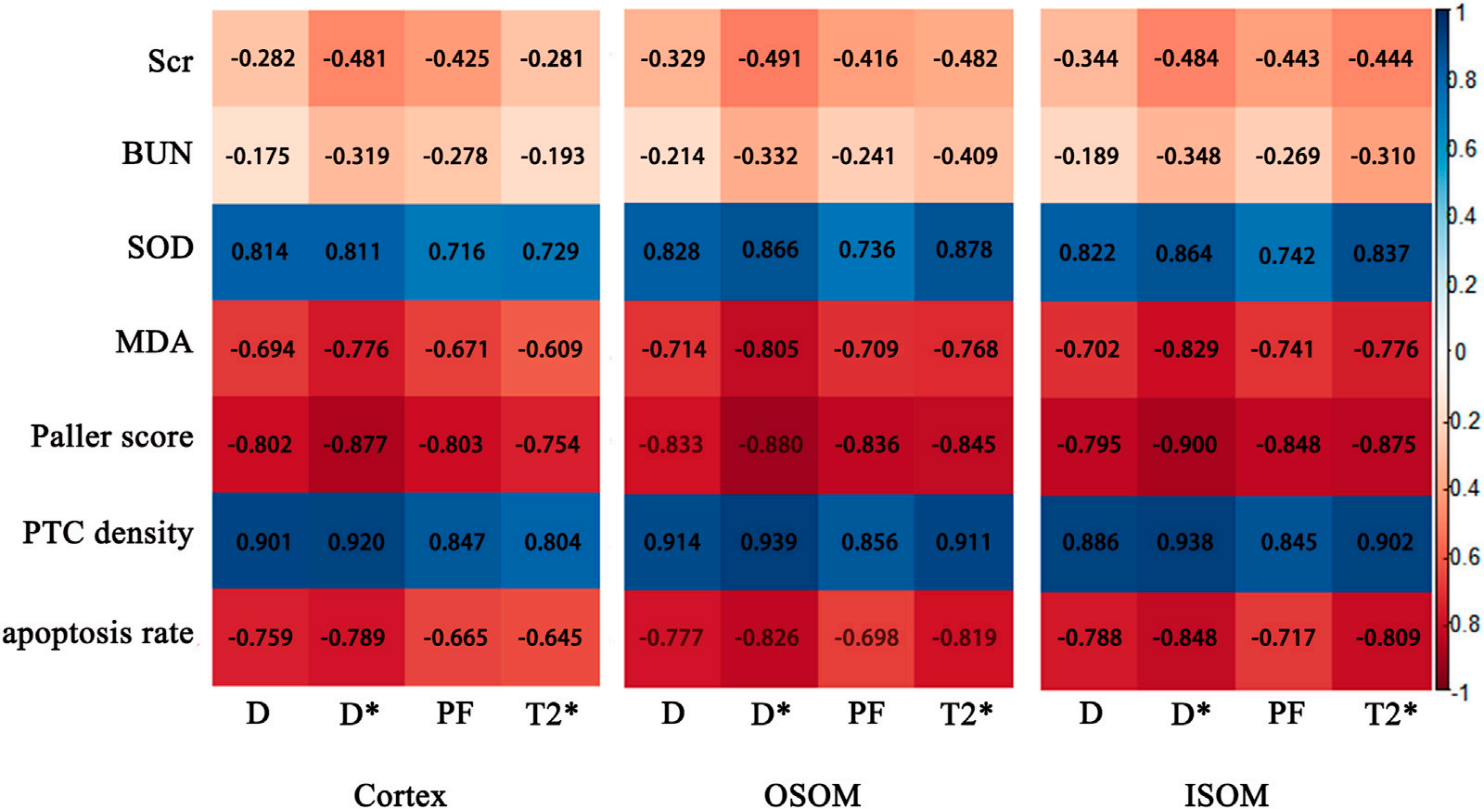
Correlation maps between IVIM, BOLD parameters of the cortex and medulla and histological, biochemical indices. Scr, serum creatinine; BUN, blood urea nitrogen; SOD, superoxide dismutase; MDA, malondialdehyde; PTC, peritubular capillary.

The correlation between MRI parameters and histopathology, blood biochemical indicators were shown in Figure 8.

## Discussion

We utilized IVIM and BOLD MRI to evaluate water molecule diffusion, microcirculation and renal oxygenation in the rat renal CIRI model with different cold ischemia times. The study demonstrated that renal D, D*, PF and T2* values showed significantly different changes and were strongly correlated with histological and immunohistochemistry changes. IVIM and BOLD parameters monitored the CIRI alternations more sensitively than the blood biochemical indicators. Thus, multiparametric MRI provided more reliable information for detecting CIRI as noninvasive and quantitative markers.

Most models utilized the WIRI model at certain time points after variable ischemic periods. However, WIRI has limited clinical relevance, as some degree of renal perfusion and oxygen consumption are continued in most clinical scenarios, with variable decline in glomerular filtration and tubular reabsorption. In fact, WIRI might be only relevant to post-cardiac resuscitation. Even aortic clamp operations or cardiac bypass surgeries with total cessation of renal bloods flow are undertaken under hypothermic conditions, and cannot be regarded as renal WIRI. Cold-preserved transplanted kidneys are subjected to protracted cold ischemia and most often associated with tubular injury. While, to a substantial extent, hypothermia reduces tubular metabolism and protects tubular segments with limited capacity to anaerobic glycolysis, transforming damage pattern from principally S3 proximal tubular segments to medullary thick limbs. Our current study expanded the scope of previous work by establishing the model of cold ischemia that the right kidney was resected completely in order to eliminate compensation effect of the contralateral kidney and simulate clinical kidney transplantation environment. The results of our study demonstrated multiparametric MRI can be a potential candidate for noninvasive assessment of renal CIRI.

The D value represents the true water molecule diffusion level (Le Bihan D et al., 1986). In our study, the D values in Group T4 were notebaly lower than those in Group T0 and T2 at the same timepoints, since cellular edema has developed and liquid flow was restricted within the lumen, resulting in limited water diffusion and significant decrease in the D value. At 1 h after CIRI, the D values of cortex and medulla in all the groups declined significantly. Combined with the pathology manifestations of 1 h, we found that damaged brush border shedding in the dilated renal tubules, inflammatory cells casting in the lumen and loss of glomerular capillary structure, which resulted in complex microstructures formation therefore the diffusion of water molecules was greatly restricted (Polichnowski et al., 2020). Our study also showed the D values began to rise significantly on day 1 and remained progressive increase then returned to the normal levels in Group T0 and T2. This D value change trend coincided with reversible renal cell injury post CIRI in pathology view (Wei et al., 2019). Feng et al. (Feng et al., 2021) reported that increased D value could be related to decreased renal tubular epithelial cell edema and reduction in the tubular flow, which was similar to our study.

The D* value reflects the microcirculation perfusion and is related to the liquid flow velocity. The kidney is an important circulatory organ of human body as its blood flow volume accounts for 25% of cardiac output. It is therefore very sensitive to CIRI (Ellison, 2021). In our study, the D* value exhibited a significant decrease at 1 h in all groups, findings consistent with a previous study (Hueper et al., 2017). The D* values did not restore to the baseline levels in Group T2 and T4 demonstrating that the microcirculation was impaired at the early stage of CIRI. Although main renal artery blood flow is reflowed, restoration of renal microvascular perfusion may remain damaged. There are mainly three mechanisms accounting for the microcirculation disorders (Smith et al., 2019). First, the renal capillary networks are compressed resulting from interstitial edema and endothelial cell swelling (Zhang B. et al., 2018). In the present study, we found that the Paller score reached to the peak on day 1 indicating that edema and swelling were the most serious degree. Second, intraluminal flow can be reduced due to generation of vascular contracting substances caused by vascular endothelium dysfunction (Francis and Baynosa, 2017). Third, the “no-reflow phenomenon” resulted from the attenuated vascular relaxation after reperfusion is characterized by increasing impedance of microvascular blood flow (Eltzschig and Eckle, 2011). Above all, the peritubular capillaries may become deformed and the amount decrease thus the D* measurement showed strong correlation with PTC density, which furtherly emphasized the potential of D* in evaluating CIRI.

The PF value represents the percentage of microcirculation blood volume and renal tubular fluid volume in the total tissue fluid volume (Eric et al., 2012). The PF value exhibited a sharp elevation on day 1 and obviously exceeded over the baseline, subsequently decreased on day 2 in Group T0. This may attribute to the compensatory hemodynamics initiation and high filtration in Group T0 on day 1. In our study, the PF measurements in the OSOM and ISOM returned to the baseline levels later than cortex in CIRI groups, suggesting that the cortex recovered quicker than medulla (Pohlmann et al., 2013). Cheung et al. (Cheung et al., 2010) found that the PF value of renal cortex significantly increased from day 1 to day 2 post ischemia - reperfusion injury. This could be possibly explained by the fact that the cortex received the majority (90%–94%) of the renal blood flow and was less susceptible to hypoperfusion and hypoxia than medulla. Liu et al. (Liu et al., 2018) also revealed that medulla was sensitive to hypoperfusion, which was similar to our results. It may be due to the fact that the medulla is nourished from efferent arterioles of deep juxtamedullary glomeruli that receives only 10% of renal blood flow, accounting for the limited oxygen supply to the medulla (Brezis M and Rosen S., 1995). In addition, our micropathology results showed that cellular debris and brush border fell off in the tubules and tube casting formed which may affect tubule fluid dynamics by increasing tubule resistance and fluid velocity, which was in line with Ebrahimi’s study (Ebrahimi et al., 2014). These may be the appropriate explanations for significant correlation between PF values and Paller score. Mao et al. (Mao et al., 2018) found that the PF value of the cortex was well correlated with the renal tubulointerstitial pathological scores, which was consistent with our study. These findings indicated that IVIM imaging could reflect progressive microstructure changes of the renal CIRI and contributed to monitoring the severity of CIRI noninvasively and quantitatively.

BOLD MRI depends on the alternation of local tissue oxygenation level. CIRI could give rise to hypoxia in kidney tissue and leaded to the increase of deoxyhemoglobin concentration which was paramagnetic and produced local magnetic field inhomogeneity, thus T2* relaxation time was shortened. Therefore, T2* value could be an important indicator to reflect the tissue hypoxia. (Zhang et al., 2021). The present study indicated that longer cold ischemia times resulted in decreased T2* values. On one hand, only 10% of the renal blood flow is distributed in the outer medulla and the thick ascending limbs of medulla is mainly responsible for the reabsorption of sodium, which is a process requiring much oxygen. On the other hand, the loss of capillaries around the tubules after CIRI surgery leads to a decrease in oxygenated hemoglobin in the blood vessels. Thus, the limited oxygen supply and high demand make the medulla more susceptible to hypoxia damage. (Zhang et al., 2021). As reperfusion time was prolonged, the T2* values increased gradually. The T2* values of the Group T0 and T2 were restored to the normal level in 2 days indicating that the intrarenal hemoxygenation level may be recovered for the ischemia time less than 2 h. Pan et al. (Pan et al., 2021a) investigated that BOLD MRI can noninvasively evaluate the changes of oxygenation in rats with susceptible renal ischemia and reperfusion injury. Zhang et al. (Zhang et al., 2019)found that the severity of the injury is related to the ischemic duration and in early AKI, which was consistent with our findings. The present study demonstrated that T2* value of the medulla in the Group T4 still declined in day 1 whereas inclined in cortex. It may be due to the fact that the blood supply and partial pressure of oxygen of the renal cortex are much more than those of the medulla. The medulla requires abundant oxygen for energy transmission thus it is vulnerable to hypoxic injury (M and S, 1995). The previous research have mentioned that ISOM might be more sensitive to CIRI and OSOM might be more sensitive to WIRI (Abassi Z, 2019; Heyman S N, 2010). Similarly, in our study, the T2* values of ISOM declined slightly more at 1 h compared to OSOM, indicating that the ISOM may be more sensitive to CIRI and suffer more severe damage for the reason that the thick ascending medullary branches demand large amounts of oxygen to support reabsorption (Heyman S N, 2010). While, there were no significant differences of MRI parameters between ISOM and OSOM, which was considered to contribute to the relatively short ischemia duration. Some literature mentioned that if the cold ischemia time was long enough to exceed 10 h, the damage to ISOM may be more obvious (Heyman S N, 2010).

Histologic analysis showed different degrees of kidney injury among sham-operated and CIRI groups. At 1 h after operation, the pathology results mainly manifested as serious loss of brush border and tubular swelling, accounting for the increased Paller score in the CIRI groups (Group T2 and T4), which were consistent with a previous research (Chang et al., 2019). However, the previous research (Pan et al., 2021b) found that the severity of the brush border destruction and tubular epithelial necrosis were highest at 48 h after reperfusion in the IRI model. We speculated that the ischemia temperature differences are responsible for this varaition. The correlation analysis showed a strong correlation of MRI parameters and Paller scores, supporting its ability to evaluate the renal tubule injury. PTC start from the glomerular outflow arterioles and mainly distributed around the renal tubules with the function of providing oxygen and energy for nephrons (Chen et al., 2020). In our study, the PTC density were dramatically reduced among these groups illustrating that CIRI may cause microcirculation dysfunction, which was consistent with the previous study (Basile et al., 2001). It was notable that D* meaurements were strongly correlated with PTC density furtherly confirmed IVIM could reflect the course of kidney injury in CIRI. Secondly, the loss of peritubular capillaries after IRI led to decreased oxygen content in the vessels. In our study, the apoptosis rate increased progressively then decreased on day 2 after CIRI. Prior studies have shown that proximal renal tubular epithelial cells are involved, the apoptotic protease are activated and metabolic disorder is induced during renal CIRI (Bertheloot et al., 2021). The present study demonstrated that the apoptosis rate reached the peak level on day 1 rather than 1 h after reperfusion, which could be explained by the Jain’s (Jain et al., 2021) research that the activation of some signal transduction pathways and expression of apoptosis-inducing gene take a period of time. Above all, the analysis results showed a strong correlation between MRI parameters and pathology indicators, supporting the ability to assess kidney function recovery after CIRI.

Our study illustrated that there were no obvious differences for Scr and BUN in Group T0 at all timepoints. However, the MRI parameters showed statistically significant difference in various groups. Meanwhile, histological images appeared as corresponding renal damage. A previous study (Wei et al., 2019) reflected that renal function showed no dominant reduction after CIRI only judged by Scr and BUN contents. This was mainly due to the strong compensatory function of the kidney and Scr exhibited abnormal only when more than half of the nephrons were impaired (Yu et al., 2021). The correlation analysis showed no strong correlation between MRI parameters and Scr, BUN. Therefore, the MRI may detect alternation more sensitively and earlier than biomarkers in the early stage of renal CIRI. The current study illustrated that the activity of SOD exhibited a sharp decline followed by gradual incline after CIRI. It may be interpreted that damaged vascular endothelial cells and renal tubular epithelial cells release a large number of reactive oxygen free radicals post CIRI (Winter et al., 2011). SOD could scavenge oxygen free radicals, repair damaged cells in time and improve the ability of antioxidant damage (Ozturk et al., 2018). MDA is a metabolite of lipid peroxidation, which leads to cell damage and forms lipid peroxide. In the Group T2 and T4, MDA elevated obviously at 1 h and continued to increased on day 2. The previous research (Cakir et al., 2017) also revealed that renal ischemia reperfusion injury led to the decrease in SOD activity while increase in MDA, which was consistent with our study.

There are several limitations in the current study. First, to achieve enough histological analysis data, the rats did not undergo dynamic examination of the MR parameters over continuous time points. Second, the small animal number of all groups was a limitation of this study. Further investigations with larger sample size are needed to confirm these results. Third, the use of anesthetics may have a certain protective effect on renal CIRI, which was difficult to completely avoid the influence. We can investigate the MRI changes of how to protect renal CIRI by different anesthetics in the future. Moreover, the observation time was not a long duration so it could not reflect long-term progression. Further studies on prolonging detection time in kidney CIRI models are necessary.

In conclusion, our study demonstrated that IVIM and BOLD are useful for monitoring kidney function impairment and recovery after CIRI. The D, D*, PF and T2* measurements were of great value reflecting that the dynamic changes of renal cell edema, microcirculation and blood oxygenation level. Histologically, these parameters were strongly correlated with the tubular injury scores, PTC density and cell apoptosis rate. In addition, MRI measurements detect the renal function alternations more sensitively than Scr and BUN at the early stage of CIRI. So IVIM and BOLD MRI can be considered as noninvasive and quantitative methods for assessing the renal microstructure and function alternations in CIRI.

## Data Availability

The original contributions presented in the study are included in the article/Supplementary Material, further inquiries can be directed to the corresponding author.
