# Supplementary material for: Evaluation of renal cold ischemia–reperfusion injury with intravoxel incoherent motion diffusion-weighted imaging and blood oxygenation level-dependent MRI in a rat model
Source: Front Physiol. 2023 May 22;14:1159741. doi: 10.3389/fphys.2023.1159741 (PMC10240072; doi:10.3389/fphys.2023.1159741)
Supplement: Supplementary file 1 [file Table1.docx]

**Supplementary Material**

**Table 1.** The ICC analysis about the IVIM parameters in all rats between two radiologists

|  |  | **ICC value** | **95% CI** |
| --- | --- | --- | --- |
| **CO** | **D** | 0.953 | 0.887-0.980 |
|  | **D*** | 0.841 | 0.505-0.940 |
|  | **PF** | 0.958 | 0.796-0.986 |
|  | **T2*** | 0.920 | 0.826-0.964 |
| **OSOM** | **D** | 0.881 | 0.750-0.946 |
|  | **D*** | 0.920 | 0.828-0.964 |
|  | **PF** | 0.880 | 0.748-0.945 |
|  | **T2*** | 0.928 | 0.845-0.968 |
| **ISOM** | **D** | 0.887 | 0.761-0.949 |
|  | **D*** | 0.868 | 0.724-0.940 |
|  | **PF** | 0.903 | 0.792-0.956 |
|  | **T2*** | 0.916 | 0.820-0.962 |

D, pure molecular diffusion; D*, pseudo-diffusion; PF, perfusion fraction

CO, cortex; OSOM, outer stripe of outer medulla; ISOM, inner stripe of outer medulla

ICC, intraclass correlation coefficient; CI, confidence interval
